# Shared Mechanisms of Chronic Pain and Emotional-Motivational Problems: From Basic Science to the Clinics

**DOI:** 10.1155/2018/9305026

**Published:** 2018-11-19

**Authors:** Susanne Becker, Edita Navratilova, Frauke Nees, Stefaan Van Damme

**Affiliations:** ^1^Department of Cognitive and Clinical Neuroscience, Central Institute of Mental Health, Medical Faculty Mannheim, Heidelberg University, Mannheim, Germany; ^2^Department of Pharmacology, University of Arizona, Tucson, AZ, USA; ^3^Department of Experimental-Clinical and Health Psychology, Ghent University, Ghent, Belgium

Chronic pain is often associated with altered emotional and motivational states. High comorbidities between chronic pain and affective disorders are a well-known phenomenon. Many overlaps in the neural processing of pain, motivation, and emotion have been reported. For example, multiple brain regions that are involved in the processing of pain are also implicated in the processing of affective, motivational, and emotional events. Similarly, dysfunction of mesolimbic circuitry, which results in impaired motivated behavior, and which has been discussed as a causal mechanism in affective disorders, has been observed in chronic pain patients. Accordingly, it has been proposed that altered functioning of brain affective and emotional circuits may contribute to pain chronification. Apart from these neurophysiological overlaps, pain, motivation, and emotion show many similarities and interactions on a psychological level. For example, pain is a typical stressor that shifts motivational systems by urging initiation of protective behaviors (e.g., escape, avoidance, and hypervigilance). In addition, positive and negative emotional experiences as well as cognitive processes can modulate the perception of pain. Well-known examples of such cognitive-emotional pain modulation are placebo and nocebo effects that can lead to inhibition and facilitation of perceived intensity and unpleasantness of pain which can be mediated by anxiety. Based on these observations, it is conceivable that the multidimensional treatments incorporating pharmacological and nonpharmacological approaches of both pain and motivational-emotional disturbances could result in fruitful synergies.

To understand such interactions and possible shared mechanisms, a multidisciplinary view on the phenomenon of chronic pain and altered emotional-motivational processing is needed. The aim of the present special issue was to provide a platform to integrate such multidisciplinary views, discussing recent advances from basic to clinical research and giving the reader insights into the intersection between chronic pain, emotion, and motivation, as well as in the mechanisms of comorbid affective disorders and possible new applications in pain therapy.

The special issue comprises eight articles providing, on the one hand, a broad overview on emotion and motivation in chronic pain together with a focus on underlying mechanisms and highlighting translational approaches. On the other hand, specific outstanding phenomena and contexts are discussed in reviews and supported by original research articles reporting novel insights. Specifically, S. Becker et al. provide a review on the current state of the art on emotional and motivational pain processing, focusing on observations suggesting that in chronic pain a shift toward negative emotional-motivational processing occurs. By discussing various factors that might contribute to such a shift, for example, altered reward processing and goal regulation, a translational view is presented, emphasizing the great potential of translational approaches. Focusing on one of the most important brain structures in the context of emotional-motivational processing, namely, the amygdala, J. M. Thompson and V. Neugebauer provide a comprehensive overview of the role of the amygdala neurocircuitry in pain, together with pain-related changes of this neurocircuitry. Importantly, possible pharmacological strategies targeting corticoamygdala dysfunction and pain-related amygdala hyperactivity are discussed from the perspective of basic and clinical science.

Three more reviews focus on phenomena related to emotional-motivational pain mechanisms pain. A.-K. Bräscher et al. review the literature on placebo hypoalgesia and nocebo hyperalgesia as forms of emotional-motivational pain modulation, concluding that the role of learning processes in this context is currently underestimated. Although the role of learning in placebo and nocebo effects is acknowledged, it appears neglected in human research. X. Fuchs et al. focus in their review on the role of emotion and cognition in phantom limb pain. Although perceptual and disability-related factors seem to be in forefront of factors affecting phantom limb pain, emotional factors, such as comorbid depression and anxiety, perceived stress, and cognitive factors, such as catastrophizing and maladaptive coping, affect severity of phantom limb pain and disability induced by the pain. Concentrating on children and adolescents with chronic pain, K. E. J. Mano delineates that anxiety is a crucial factor in paediatric chronic pain, with a comorbidity higher than in adults with chronic pain. They identify school as the major source of anxiety in children with chronic pain and discuss possible shared mechanisms of school anxiety and chronic pain as well as current approaches of assessing school anxiety.

Focusing on paediatric chronic pain as well, M. Pavlova et al. provide original data investigating the role of anxiety and depression in the relationships of chronic pain and sleep disturbances in young chronic pain patients (8–18 years of age). Paediatric pain is often associated with sleep problems, and the authors of this article show that this relationship is mediated by depressive and anxiety symptoms.

Two additional research articles investigated the role of affective-motivational and cognitive processes in physical functioning in chronic pain patients. R. Esteve et al. demonstrated that activity patterns in patients with chronic musculoskeletal pain are affected by patients' self-regulation of goals. Specifically, optimism affects this relationship, with higher levels of optimism being related to persistence, flexible goal management, and commitment to new goals, which in turn are associated with higher positive affect, persistence in finishing tasks despite pain, and less avoidance behavior. Last, O. Rasouli et al. hypothesized that higher cognitive load interferes with motor control, namely, postural control, in patients with fibromyalgia and chronic fatigue syndrome. Although this hypothesis could not be confirmed, both groups of patients displayed impaired postural control compared to healthy participants, and this impairment was correlated with perceiving fatigue related to their disorder.

In conclusion, this special issue highlights the complexity of chronic pain conditions and discusses the contribution of emotional, motivational, and cognitive factors. Understanding overlapping neural mechanisms that promote chronic pain will lead to improved prevention and treatment of chronic pain.

